# *Toxocara* Seroprevalence in Europe and Considerations for Future Research

**DOI:** 10.3390/pathogens14111117

**Published:** 2025-11-03

**Authors:** Roxana Gabriela Cobzaru, Carmen Valerica Ripa, Ramona Gabriela Ursu, Miruna Raluca Ripa, Costin Damian, Luminita Smaranda Iancu

**Affiliations:** 1 Grigore T. Popa University of Medicine and Pharmacy Iasi, 700115 Iași, Romania; 2 “Cuza-Vodă” Clinical Hospital of Obstetrics and Gynecology, Strada Cuza Vodă 34, 700038 Iași, Romania

**Keywords:** *Toxocara*, toxocarosis, parasite, molecular detection assay

## Abstract

Toxocarosis is one of the most widespread zoonotic parasitic diseases, caused by *Toxocara canis* and *Toxocara cati*. Studying the epidemiological situation is a real challenge for specialists in the field worldwide. The aim of this study was to highlight the epidemiological aspects of the prevalence rate in Europe, and we found that the distribution of *Toxocara* infection is uneven, depending on geographical, climatic and socio-economic factors. Currently, there is a continuous spread of this parasite in many regions, including economically developed countries, but the exact prevalence is not known because there are no regular reports and statistical evidence regarding toxocarosis. The presence of nonspecific symptoms and subclinical disease may delay diagnosis and treatment, with long-term implications for the patient, and because of this, we discussed the possible *Toxocara* detection methods, with several novel and interesting approaches. Toxocarosis is included among the neglected parasitic diseases, requiring seroprevalence studies to be carried out to develop programs to reduce the frequency of this parasitosis.

## 1. Introduction

Human toxocarosis (HT) is a parasitic infection mainly caused by larvae of *Toxocara canis* or *Toxocara cati* [1], which are intestinal ascarid nematodes of canids and felids, respectively [2]. The definitive hosts are mainly dogs and cats but also other canids in which the life cycle of the parasite is completed [3,4]. After ingestion of embryonated *Toxocara* eggs, containing infective third-stage larvae, via contaminated soil, water or food or uptake of larvae via raw or undercooked meat of paratenic hosts, e.g., chicken, the larvae penetrate the intestinal wall and are transported to different tissues via the blood stream [5]. It is a worldwide zoonosis affecting mainly children but also adults, and it is often asymptomatic and difficult to diagnose and treat, either because of therapeutic failure or because of the increased risk of reinfection [6].

During the course of this parasitosis, serious syndromes can occur, known as larva migrans visceralis, the most common form, larva migrans ocularis, neurotoxocarosis and subclinical toxocarosis [1,7,8,9].

In some cases, the symptoms may range from fever to granulomatous hepatitis, nephritis and arthritis, asthma and pulmonary fibrosis [10], depending on the parasite load and the age of the host. Diagnosis is generally based on the patient’s medical history, clinical examination and laboratory investigations that may reveal the presence of specific antibodies against *T. canis* [2,11,12] or detect parasite DNA in tissue samples [13,14].

*T. canis* is naturally hosted by dogs, which become infected by ingesting embryonated eggs containing third-stage larvae (L3) or paratenic hosts with infective larvae. Alternatively, offspring may be infected transplacentally during pregnancy or by trans-mammary infection via milk from dams. The increased risk of infection and its spread are a consequence of massive soil contamination with *Toxocara* eggs [15,16,17]. On the other hand, studies have reported that direct contact with the fur of cats infected with *T. cati* is another route of transmission, since potentially infective embryonated eggs have been identified in perianal areas, extremities and the lower part of the tail of cats [18,19]. Cats play a crucial role globally as the primary hosts for *Toxocara*, releasing eggs into the environment and thereby heightening public health concerns. Health authorities and cat caregivers must prioritise efforts toward preventing and managing this zoonotic disease in feline populations. This is especially crucial in regions with elevated risk factors and prevalence rates, necessitating heightened vigilance and proactive measures [20].

Human infection is achieved by ingestion of embryonated *Toxocara* eggs from soil contaminated with animal faeces. Humans are an accidental host of the parasite (intermediate host). The most vulnerable to this parasitosis are children who frequent playgrounds due to contact with soil and lack of hygiene. They can become infected by geophagy or by eating fruit and vegetables contaminated with embryonated eggs [11,21].

Immunological diagnosis allows the identification of IgG and IgE antibodies against *Toxocara* using the enzyme-linked immunosorbent assay (ELISA) method, which requires confirmation by the Western blot assay, which has a higher specificity [22,23,24].

Toxocarosis is treated with anthelmintics. The criteria for evaluating the efficacy of treatment are the progressive decrease in eosinophilia, regression of clinical manifestations and reduction in specific antibody titres [25].

In terms of prophylaxis, there is a need for permanent collaboration between the Public Health Directorate, the Veterinary Health Directorate and local authorities to develop protocols that include health education programmes aimed at reducing the factors that favour the occurrence of this infection.

The aims of this study were (1) to analyse the trend of *Toxocara* infections in the last 10 years in Europe and (2) to assess the available molecular-based *Toxocara* detection methods.

## 2. Materials and Methods

Articles were selected from the following databases: PubMed, Google Scholar, Scopus and Web of Science. The search terms used were “*Toxocara* infection humans Europe OR Human Toxocarosis Europe” in the last 10 years. The following selection criteria were followed: scientific articles published in international medical journals, studies with a publication date between October 2025 and December 2015, studies that were conducted on human subjects, studies with no age limit for the target population and studies that reported the prevalence or incidence of *Toxocara* infections. The exclusion criteria used were articles that were duplicates, editorials or articles without information on the prevalence of toxocarosis in Europe and studies referring to animal infection without relation to human toxocarosis. We excluded papers that tested soil or animal samples for *Toxocara* spp. From the selected articles, we were able to extract data on study region, sample size, total number of individuals with toxocarosis and variables (risk factors, symptoms, morbidity, socio-demographic characteristics). The results are summarised in Table 1.

## 3. Results and Discussion

Our search retrieved 87 results, of which 30 were suitable for our analysis. There were case presentation studies, retrospective studies, comparative studies, cross-sectional analyses, systematic reviews and meta-analyses. In the 30 papers that we included, the main detection assay used was ELISA, followed by Western blot confirmation. *Toxocara* studies have been published in all the European countries, with seroprevalence variations from 0.93 to 36%. One study was based on the discharge ICD code diagnosis from a nationwide database. A few studies evaluated the level of knowledge about *Toxocara* infections using questionnaires for dog/cat owners. Other studies evaluated the risk of food-borne transmission of *Toxocara* spp. to humans. Some authors tried to associate Toxocarosis with other conditions, such as significant eosinophilia, double lung transplantation, idiopathic myelitis, long-term travellers, uveitis, clinically isolated syndrome (CIS) and multiple sclerosis (MS). A high seroprevalence was detected in children with early mental health difficulties, transplantation, a lower level of education, a younger age, a male gender, consumption of undercooked or raw poultry, contact with soil and increased risks of foodborne transmission of zoonotic toxocarosis. The comparative studies identified a decrease in overall *Toxocara* seroprevalence. These results indicate the need for integrated public health interventions targeting parasitic infections in susceptible populations.

The majority of patients included in these studies were children, with age being a risk factor in association with geophagia and frequenting playgrounds contaminated with dog and cat faeces. In the analysed studies, Toxocara infection was more frequently encountered in children from rural areas who came into contact with infected animals (dogs, cats) or with contaminated soil due to activities carried out outdoors without following adequate hygiene rules.

The frequent association of toxocarosis with pulmonary dysfunction and cognitive and ocular disorders calls for a multidisciplinary approach to reduce the number of cases in the general population.

It is necessary to prioritise prevention efforts through health education activities, providing information on zoonoses, their transmission and risk factors. Limiting soil contamination with dog and cat faeces, prophylactic anthelmintic treatment and hand washing can be important factors in minimising exposure to this parasite.

Toxocarosis is among the five neglected parasitic diseases without being recognised as a major public health concern requiring new methods of diagnosis and treatment [56,57,58].

The limitations of this study are due to the lack of data on toxocarosis for a large number of countries in the European area and the fact that the available studies had limited information on the gender and age of the patients, contact with pets as well as geographical and socio-economic data.

## 4. Toxocarosis Diagnosis Methods: Future Directions

Establishing an optimised diagnostic method for toxocarosis in humans is challenging due to the nonspecific clinical symptoms and the possibility that other parasitic and viral infections may interfere with the test result.

The present method of immunodiagnosis focuses on using ELISA with excretory–secretory antigens (ES Ag) obtained from *T. canis* larvae. This approach exhibits adequate sensitivity but may produce false positive results in the presence of infections caused by other helminths, including nematodes such as *Ascaris* spp., *Enterobius* spp., *Trichuris* spp. and *Anisakis* spp.; cestodes such as *Taenia* spp. and *Echinococcus* spp.; and trematodes such as *Fasciola* spp. and *Schistosoma* spp. [59]. In order to address this issue, a Western blot technique (WB) was developed over 30 years ago, which is currently employed in a standard two-tier technique used by specialised laboratories to confirm positive ELISA results [59].

*Toxocara* does not reach the adult stage in the human host; hence, its eggs cannot be found in faeces. Classical parasitological microscopic investigations are not effective in diagnosing human toxocarosis. Due to the distribution of and scarcity of tissue-dwelling larvae in the majority of toxocarosis patients, only rarely can a conclusive diagnosis be made by histological examination of biopsies taken from affected tissues, confirming the presence of *Toxocara* larvae [60]. Nevertheless, biopsies are generally not conducted solely for diagnostic purposes due to the low likelihood of obtaining tissue that contains a *Toxocara* larva and because of its invasive and time-consuming nature. Distinguishing the *Toxocara* larvae from larvae of other ascarids can be challenging due to their physical similarities, particularly when only fragments of the larvae or larval debris are present in the tissues [61]. Occasionally, ophthalmoscopy may reveal live larvae in certain patients with an eye infection, although this is not typical. Due to the constraints in viewing and distinguishing *Toxocara*, the diagnosis of toxocarosis is typically achieved by immunodiagnostic procedures [60].

If there are elevated levels of serum anti-*Toxocara* antibodies, combined with symptoms, an increased eosinophil count and no other plausible explanation, it is highly likely that the individual has toxocarosis. Nevertheless, if there is well-documented seroconversion, the diagnosis can be regarded as conclusive. Several serological assays have been created to identify *Toxocara* infections, with enzyme-linked immunosorbent assays (ELISA) being the favoured technique for detecting the disease and conducting epidemiological studies. Immunoblot is more sensitive in detecting the presence of toxocarosis antibodies in chronic pruriginous skin conditions [61].

Studies focusing on immunoassays that target IgE against *Toxocara* have received considerable interest, especially in patients with a notable level of specific IgE, which diminishes following anti-helminthic treatment. Scientists have investigated novel methods, assay configurations and improved antigens to enhance the diagnostic accuracy and precision of the assay. Currently, the indirect IgG-based ELISA is regarded as a reliable technique for the diagnosis of human toxocarosis. Dot-ELISA, a dot-based test, demonstrated superior stability, reduced cost and ease of execution without the need for specialist equipment. Western blotting is frequently employed for diagnostic purposes, particularly to validate ELISA findings and diagnose toxocarosis in individuals suffering from chronic urticaria [61].

Molecular approaches exhibit high analytical specificity and offer faster response times compared to alternative diagnostic methods. PCR-based tests utilising diverse genetic markers have been created, allowing for the identification and phylogenetic study of *T. canis* and *T. cati*. PCR-based testing has been used to detect *T. canis* larvae obtained from human biopsies in cases of ocular larva migrans and from cerebrospinal fluid [62]. These tests, such as quantitative real-time PCR (qPCR), have been employed to precisely identify and diagnose *Toxocara* samples obtained from animal faeces or soil. Optimised molecular techniques have the capacity to enhance the diagnosis of toxocarosis [62].

### Possible Diagnostic Methods of Toxocara Infections

We aimed to analyse the worldwide proportion in which molecular biology was used for the diagnosis of toxocarosis in the last 5 years. We used as key words “*Toxocara canis* molecular detection assay”, and our search retrieved 27 results in PubMed, from which only 13 fulfilled our criteria. We excluded the ones that referred to other parasites. From the 13 studies, three used ELISA for antibody detection, so we analysed only 10 papers published in the last 5 years. We identified studies that used phages for gene sequences and binding characteristics, miRNA 21 and miRNA 103a technology, loop-mediated isothermal amplification assays and quantitative real-time polymerase chain reactions. The authors used laboratory animals for their experimental research and aimed to find a sensitive assay for the optimal detection of *T. canis* [63,64,65,66,67,68,69,70,71]. Our findings are described in Table 2.

The current two-stepped immunodiagnosis technique requires a greater amount of time than a simple ELISA [72], which makes it significantly more expensive, even unaffordable for certain diagnostic facilities and low-resource areas. A more advanced ELISA with improved specificity is necessary, and as a response to this need, several research teams have obtained recombinant antigens from particular fractions within the ES Ag with a low molecular weight and employed them in ELISA assays [73]. Experimental research has reported that ELISA utilising recombinant antigens has been said to possess comparable sensitivity to ES Ag ELISA while also demonstrating high specificity [74]. Nevertheless, this research is still new, there is a lack of information on the regular utilisation of these ELISA tests, and there are currently no commercially accessible kits.

The present immunodiagnostic approach has an additional constraint, which is the lack of the ability to differentiate between a previous infection and a current, active *Toxocara* infection. Following this direction, a method was employed to evaluate the avidity of anti-ES Ag antibodies [75]. This approach has demonstrated its effectiveness, and there are commercially accessible kits, although still lacking uniformity [76]. Nevertheless, this method necessitates an additional stage in the immunodiagnostic process, which increases the turnaround time and costs, probably the reason why the evaluation of avidity has not been widely adopted. One potential future possibility is the identification of ES Ag in the blood of subjects who have a positive immunodiagnostic result. However, most cases of *Toxocara* infections are mild/covert, characterised by a low parasite load. Several results of experimental research using sandwich-capture ELISA with nanoantibodies are presented in Table 1 [64,66,68].

Regarding the molecular detection of helminthiases, the identification of soluble DNA has proven to be effective in diagnosing different types of filariases and schistosomiasis [77], infections in which the parasite is present in the bloodstream or lymphatic system. Currently, molecular detection is performed by specialist laboratories using various methodologies and specific primers. In the case of toxocarosis, molecular approaches are particularly useful for identifying the larvae in different organ biopsy samples [63]. Most often, ocular- and neurotoxocarosis occur when a single larva of the *Toxocara* spp. parasite is found in a confined area like the eye or central nervous system. To diagnose ocular toxocarosis, the ELISA method is used to identify antibodies against *Toxocara* in the aqueous humor [78]. Even though this method is satisfactory for about 50% of ocular toxocarosis cases, the problem becomes more complex when specific antibodies are present in the blood due to the coexistence of widespread toxocarosis. Consequently, specialist laboratories identify soluble *Toxocara* DNA in aqueous or vitreous fluids and, occasionally, in cerebrospinal fluid [78]. Data regarding these conditions in Europe has not been published due to the rarity of these cases.

Our review has some limitations regarding the “recruitment biases”, concerning the prevalence of toxocarosis detected in our analysed papers: Selection Bias and Detection Bias. Selection Bias includes volunteer bias (individuals who volunteer for testing or surveys may have different health behaviours or risk factors compared to the general population) and convenience sampling: using samples that are easy to collect, such as people in a particular location or at a specific event, might not represent the broader population. Detection Bias refers to Differential Testing (if certain groups are more likely to be tested (e.g., symptomatic individuals or those with known exposure)) and Access to Healthcare (people with better access to healthcare services are more likely to be tested and diagnosed). The analysed studies were scarce and very heterogenous in their methodology, inclusion and exclusion criteria and diagnostic assay used.

There is a need for the identification of novel biomarkers for the diagnosis of active human toxocarosis, for differential diagnosis and for detecting the parasite when it is present in very small amounts. Molecular assays are more sensitive than antibody detection, and these highly sensitive methods could be employed in the future for the diagnosis of this parasitic disease.

## 5. Conclusions

Although often asymptomatic, toxocarosis is an important health problem in Europe and globally due to visceral, neurological and ocular damage. The aim of this study was to systematise the available data on the prevalence of toxocarosis in Europe. It is very important to underline that there is a difference between *Toxocara* infections within a population on one hand and the prevalence of the disease toxocarosis on the other.

In this paper, we analysed the trend of *Toxocara* infections in the last 10 years in Europe, and we assessed the available molecular-based *Toxocara* detection methods. The comparative studies identified a decrease in overall *Toxocara* seroprevalence. A high seroprevalence was detected in children with early mental health difficulties, transplantation, a lower level of education, a younger age, a male gender, consumption of undercooked or raw poultry, contact with soil and increased risks of foodborne transmission of zoonotic toxocarosis.

As molecular methods for *Toxocara* detection, we mention phages for gene sequences and binding characteristics, miRNA 21 and miRNA 103a technology, loop-mediated isothermal amplification assays and quantitative real-time polymerase chain reactions.

Considering the progressive impact of this parasite on the population, it is necessary to improve public health policies by developing protocols including diagnostic and treatment techniques. To obtain information that allows inter-regional comparisons and reproducible results, standardised methods with high accuracy and performance are required, with the molecular detection assays being more sensitive than antibody detection.

## Figures and Tables

**Table 1:** *Toxocara* seroprevalence in Europe.

No.	Author, Year, Country, Reference	Sample Type and Size	Assay	Toxocarosis Prevalence/Related Data	Clinical Utility
1	Kanecki K, 2025, Poland, [26]	ICD-10 code diagnoses in a nationwide database 3559 patients—1888 males (53%) and 1671 females (47%)	Surveying the database for diagnosis of visceral larva migrans (VLM, toxocarosis; ICD-10 code B83.0).	The mean VLM hospitalisation rate was estimated at 0.93 per 100,000 admissions.	This retrospective study reported data that may be useful for comparative analyses in global contexts. The authors underlined the importance of higher public health standards for the monitoring and prevention of VLM.
2	Fiľakovská Bobáková D et al., 2025, Slovakia, [27]	88 blood samples from children 49 children from the Slovak majority population and 39 from marginalised Roma communities (MRCs)	Anti-*T. canis* IgG antibodies were detected in serum samples using ELISA.	The children belonging to the majority population had a seropositivity rate of 20.4%, while children from MRCs had a higher detected prevalence of 35.9%.	This comparative study found a higher prevalence of toxocarosis in MRCs, and *Toxocara* seropositivity was associated with higher levels of early mental health difficulties. Integrated public health interventions for parasitic infections are especially necessary in susceptible populations.
3	Ardelean AA et al., 2025, Romania, [28]	Serum samples from 1347 Romanian blood donors	Anti-Toxocara canis IgG antibodies were detected in serum samples with an ELISA IgG kit (Euroimmun, Germany, Berlin), using the EUROIMMUN Analyzer I-2P.	Anti-*Toxocara* antibodies were detected in 29.6% of samples. A higher seroprevalence was observed in donors from rural areas compared to urban areas and in male donors compared to females.	The authors used a multivariate logistic regression model, which indicated that the main factors associated with a higher *Toxocara* seroprevalence were a lower level of education, younger age, male gender, consumption of undercooked or raw poultry, and contact with soil. *Toxocara* infection prevalence in this region was considered significant, underlining the necessity of health education programs that focus on public awareness and promote preventive behaviours in populations with the identified risk factors.
4	Healy S et al., 2025, UK, [29]	1000 computer modelled samples of spinach and meat	The models were based on previous field data for spinach estimated egg load by qPCR and meat seroprevalence data for larval load estimated by ELISA. The mathematical models were created in a Bayesian framework.	The average human risk of *Toxocara* spp. infection per portion of spinach consumed was estimated as 0.016%, and for undercooked meat, the estimated risk was 0.172% per portion.	The models estimated a low risk of infection with *Toxocara* spp. by consuming these foods, although with a 10-fold higher infection risk in the case of consuming undercooked meat compared to spinach.
5	Reis J et al., 2025, Portugal [30]	A clinical report of a 56-year-old woman, with persistent significant eosinophilia and elevated serum IgE levels	Significant eosinophilia (5.52 × 10^3^/μL) and elevated serum IgE levels (19,253.00 IKU/L) were determined. Anti-*Toxocara* IgG antibodies were tested by ELISA and immunoblot methods.	Serological testing for *Toxocara* returned positive results by both methods.	Toxocarosis was suspected after the patient was diagnosed with Pica disorder. This study highlights the importance of considering parasitosis as a cause for eosinophilia and elevated IgE levels, even in the absence of symptoms.
6	Healy SR et al., 2024, UK, [31]	266 muscle or liver samples from 155 different food-producing animals in the south, southwest and east of England	Anti-*Toxocara* antibodies using a commercial ELISA kit. Microscopic examination of the sample for detecting larvae.	The overall prevalence of anti-*Toxocara* antibodies in tissue exudates was 27.7%. No larvae were observed microscopically in any of the samples.	The high seroprevalence in tissue exudates suggests food animals are commonly exposed to this parasite in England and supports this testing on meat products within the human food chain in support of food safety.
7	Bustamante J et al., 2022, Spain, [32]	Serum samples from 931 migrant children	IgG anti-*T. canis* antibodies were tested using an ELISA commercial kit (NovaLisa, NovaTec Immundiagnostica GmbH, Germany, Berlin).	49 children (5.3%) were seropositive.	Toxocarosis is commonly asymptomatic in children, and eosinophilia is not always present. Serological tests should be included in migrant health screening and in the diagnostic assessment of eosinophilia.
8	Balacheff Q et al., 2021, France [33]	A clinical report of a 69-year-old Caucasian man who underwent a double lung transplantation in August 2018 for idiopathic pulmonary fibrosis	Anti-*Toxocara* IgG detection was carried out using an ELISA commercial assay (NovaLisa, NovaTec Immunodiagnostica GmbH, Germany) and a Western blot assay (LDBIO Diagnostics, France, Paris).	The ELISA test returned a negative result, but the Western blot test showed a typical positive profile.	The diagnosis was established based on the Western blot test result, as it had higher sensitivity and could detect a low antibody titre, due to low larval tissue presence. The authors consider that transplanted patients should be educated with a focus on the contamination through ingestion of uncooked paratenic host meat (rabbit, chicken) or interactions with the environment and the importance of hand hygiene.
9	Fecková M et al., 2020, Slovakia, [34]	Serum samples from 1489 volunteers with no symptoms of acute disease	IgG anti-*Toxocara* antibodies were detected by ELISA.	The overall seropositivity to *Toxocara* was 3.7%. The highest seropositivity rate was detected in Roma youth/children (40.3%), followed by farmers (5.5%) and hunters (5.1%), and the lowest rate, under 1%, was in veterinarians.	The results underline the importance of preventive measures and the need for improving the knowledge of toxocarosis among professionals as well as the public.
10	Kantarakia C et al., 2020, Greece, [35]	Questionnaire answers from 185 respondents	The objective was to assess the level of knowledge about echinococcosis and toxocarosis among cat/dog owners compared to non-pet owners.	Awareness related to the risk of *Toxocara* egg contamination was limited, with no difference between owners and non-owners.	The authors support the need for public health measures in the Mediterranean region regarding education about these zoonoses in order to limit their transmission.
11	Macejova Z et al., 2020, Slovakia, [36]	Data from a cross-sectional study that included 452 Roma and 403 non-Roma subjects from Slovakia	Anti-*T. canis* IgG antibodies were detected in serum samples using ELISA.	Positive serology was more common in the Roma group (22.1%) compared to the non-Roma group (1%).	The authors conclude that lower socio-economic conditions, an unhealthy lifestyle and limited access to healthcare are factors that affect the Roma population in segregated settlements and lead to an increased prevalence of some parasitic diseases.
12	Pennelegion C et al., UK, [37]	Questionnaire answers from 500 dog owners and 500 cat owners	The objective was to assess the rate of deworming in pets according to their risk of contracting and transmitting parasitic diseases.	The average number of dewormings per year was 3.1 for dogs and 3.1 for cats, below the minimum 4 recommended.	This study indicated that both felines and canines received lower than recommended dosing frequencies in order to reduce the risk for *Toxocara* spp. egg-shedding and to improve overall pet health. Also, pet owners should be educated to assess their pet’s risk and adhere better to deworming guidelines.
13	Skulinova K et al., 2020, Czechia, [38]	Serum samples from 4428 patients were tested in a national reference laboratory	The samples were tested for anti-*Toxocara* IgG antibodies.	160 (3.6%) individuals included in the study were seropositive for *Toxocara*.	This study showed a decrease in overall Toxocara seroprevalence in the Czech population compared to data from 1998 and 2004.
14	Magnaval JF et al., 2020, France, [39]	106 file records of patients diagnosed active, symptomatic, common/covert toxocarosis	The patients were divided into two groups—atopic (49) and non-atopic (57)—and serum total IgE and specific anti-*Toxocara* IgE were compared.	The study found no statistically significant difference in the seroprevalence of the two groups.	The authors reported that atopy did not influence the clinical or laboratory pictures of patients with toxocarosis.
15	Strube C et al., 2020, Germany, [40]	41 European publications were analysed to establish an overall *Toxocara* seroprevalence in Europe	A meta-analysis of the studies was performed.	The overall seroprevalence was determined to be 6.2%, and a decade-by-decade analysis from the 1970s to 2010s showed an increase from 1.6% to 12.4%.	The authors pointed out that the apparent increase in prevalence could have been due to the increase in test sensitivity and study design biases. The research group underlined the importance of a One Health approach for toxocarosis management.
16	Nicoletti A et al., 2020, Italy, [41]	Cerebrospinal fluid (CSF) samples from 28 patients with idiopathic myelitis	Antibodies against *Toxocara* spp. were measured in CSF using a multiplex bead-based assay and an immunoblot assay.	All samples tested negative for the presence of anti-*Toxocara* IgG antibodies.	This study found no evidence of neurotoxocarosis contributing to the burden of myelitis.
17	Brydak-Godowska J et al., 2018, Poland, [42]	Medical records of 279 patients diagnosed with uveitis were analysed	Statistical analysis of the medical records also evaluated the aetiology of uveitis.	Toxocarosis was fond to be the cause for uveitis in 6.1% of cases, with a statistical difference between male and female patients.	This paper showed that the causes of uveitis in Europe are varied and that genetic, geographic, social and environmental factors could influence its cause in different populations.
18	Peju M et al., 208, France, [43]	Medical records of 298 patients with eosinophilia were analysed	Statistical analysis of the medical records evaluated the aetiology of eosinophilia.	91 patients were tested for helminthic infections, and 3 were diagnosed with toxocarosis, all of whom did not travel to a tropical area.	Drug-related eosinophilia was found to be the principal aetiology. In eosinophilic patients who did not travel to a tropical region, the authors recommend that toxocarosis should be the only helminthosis tested as aetiology.
19	Overbosch FW et al., 2018, the Netherlands, [44]	Pre- and post-travel blood samples from 604 long-term travellers	IgG antibodies against *Toxacara* and various other parasites were tested by different commercial ELISA kits.	Antibodies against *Toxocara* spp. were detected in 1 patient (0.2%).	Long-term travellers were considered to have a low risk of helminthic infections, and routine screening for eosinophilia appeared not to be of diagnostic value.
20	Kroten A et al., 2018, Poland [45]	Blood samples and medical data were analysed for a cohort of 66 children with toxocarosis over 24 months	The toxocarosis diagnosis was based on specific IgG antibody detection by ELISA and Western blot. Total IgE titres were determined by IF.	School children had higher IgG titres than preschoolers, and their tier significantly decreased after treatment. Total IgE concentrations were increased in 31 of 55 (56%) tested children.	Children at risk for toxocarosis were found to have poor hygiene habits and daily contact with dogs, and they were at risk of reinfection and limited treatment efficiency. The authors advised doctors to suspect toxocarosis, particularly in patients from areas heavily contaminated with *Toxocara* eggs.
21	Boldiš V et al., 2018, Slovakia [46]	Serum samples from 62 patients suspected of toxocarosis	Serum samples were tested for specific IgG and IgA antibodies by ELISA, followed by an IgG avidity test.	All 52 patients were seropositive for anti-*Toxocara* IgG. Of these, 32.7% were also IgA seropositive, found to be highest in the oldest age groups (*p* = 0.026).	The authors considered that anti-*Toxocara* IgA can be tested to facilitate the diagnosis of toxocarosis in acute infection. This should be correlated with other immunological markers, such as increased total IgE, eosinophilia and low-avidity IgG antibodies.
22	Jõgi NO et al., 2018, Norway, [47]	Paired medical data and blood samples from 171 parents and 264 children	IgG anti-Toxocara antibodies were detected by ELISA.	The specific antibodies were detected in 17.5% of patients and 8.0% of offspring.	This study indicated that parental *Toxocara* seropositivity was associated with increased offspring allergies in a sex-specific pattern. Exposure to this helminth was more frequent in parents than children, but seropositivity was associated with increased risk of allergies in offspring but not among parents.
23	Posová H et al., 2017, Czechia [48]	Blood samples from 220 patients with clinically isolated syndrome (CIS) and 62 multiple sclerosis (MS) patients	Antibodies against *Toxocara* were measured with an ELISA method together with eosinophil count and specific antibodies against other helminths.	21 CIS patients showed eosinophilia, but none were seropositive for *Toxocara*. All MS patients had normal eosinophil levels, and 1 (1.6%) MS patient had a positive serological result.	The authors considered that *Toxocara* infection did not represent a potential trigger of MS and that the study indirectly confirmed that parasitic infections may be protective against autoimmune disease.
24	Martelli G et al., 2017, Italy, [49]	Blood samples from a total of 930 adult immigrants were investigated for 5 neglected parasitic diseases	Individuals with a high eosinophil count were tested for *Toxocara* serology using an ELISA test (‘DRG *Toxocara canis* ELISA’, DRG Instruments GmbH, Germany).	Seroprevalence for *Toxocara* was found to be 9.7% (11/113).	This study emphasised that neglected tropical diseases pose a major health issue among immigrants and underlined the need for targeted public health action.
25	Gabrielli S et al., 2017, Serbia, [50]	Serum samples from 40 children and 298 adults	Specific anti-*Toxocara* antibodies were detected by ELISA, followed by Western blot confirmation.	The overall prevalence detected by ELISA was 23.5%, and 13.0% of the examined population was confirmed to be positive by Western blot.	The authors found the sensitivity of the ELISA test to be 100%, but with a specificity of 63.8%, and recommended that all laboratories follow a two-step diagnostic approach.
26	Halsby K et al., 2017, UK, [51]	Medical records of 127 patients, diagnosed positive for toxocarosis during 2000–2009, from a hospital of tropical diseases and 672 positive patients from a surveillance database	Specific antibodies were determined by ELISA only, during the period 2000–2005, followed by the introduction of Western blot confirmation since 2005.	The prevalence was high during the period 1983–1996, with an important decrease in the period 2000–2009.	The authors suggest that public health campaigns in reducing environmental contamination might have been the cause of the decrease in the number of cases.
27	Lassen B et al., 2016, Estonia [52]	Serum samples from 999 adults representing general Estonian population Estonian population and its selected subgroups for serological evidence of exposure to *Ascaris lumbricoides*, *Echinococcus* spp., *Taenia solium* and *T. canis*	IgG antibodies against *T. canis* and other parasites were detected by a commercial ELISA assay, followed by Western blot confirmation.	*T. canis* seroprevalence was 12.1%, and *Toxocara* spp. seroprevalence was 14.5.	Confirmed *Toxocara* spp. seroprevalence was found to be higher in animal caretakers than in the general population. Exposure to zoonotic parasites was found in all tested groups. The authors underline the need for higher awareness of zoonotic parasitic infections in Estonia.
28	Mughini-Gras L et al., 2016, The Netherlands [53]	Serum samples from 1159 individuals collected in 1995–1996 and from 3675 individuals collected in 2006–2007	Anti-*Toxocara* and anti-*Ascaris* IgG antibodies were detected using an ELISA assay.	*Toxocara* seroprevalence decreased significantly from 10.7% to 8.0%, while *Ascaris* seroprevalence increased significantly from 30.4% to 41.6% during the 10-year period.	Differing trends in *Toxocara* and *Ascaris* seroprevalence were observed, attributed to improved pet hygiene and increased exposure to pig-manure-contaminated soil. This study suggests that these infections are mainly environmentally transmitted, with contact with contaminated soil and ownership of cats or pigs representing key modifiable risk factors.
29	Papavasilopoulos V et al., 2016, Greece [54]	270 soil samples around Athens and 25 blood samples from pregnant women living in the area	Microscopy of the soil samples was performed to detect eggs. An ELISA IgG assay was used for the serum.	The prevalence of *T. canis* infection in a population of Greek pregnant women was found to be at a rate of 17.16%. Soil sample positivity rate was 17.08%.	The authors suggest a correlation between the positive response in the ELISA assay IgG antibodies and the activities of people where soil was contaminated by *Toxocara* eggs.
30	Boldiš V et al., 2015, Slovakia, [55]	Serum samples from 7678 individuals	Anti-*Toxocara* IgG antibodies and IgG avidity were evaluated by an ELISA assay.	The IgG anti-*Toxocara* seroprevalence in people from western Slovakia was found to be 15.3%, higher in the oldest age groups. Low-avidity anti-*Toxocara* IgG antibodies were detected in 27 cases out of 88 and were associated with eosinophilia.	The study indicated that, besides anti-*Toxocara* IgG, the measurement of IgG avidity could be a useful test for acute toxocarosis and should be correlated with other determinants of examined patients, such as eosinophilia, increased total IgE and age.

**Table 2:** The serological and molecular methods used to detect *Toxocara* spp.

Author, Year, Country	Aim of the Study and Assay Used	Results	Clinical Utility
Serological methods			
Baharudeen Z et al., 2022, Malaysia [63]	The authors aimed to isolate and produce novel recombinant monoclonal antibodies against *T. canis* recombinant TES-26 antigen (rTES-26) by utilising a human helminth scFv phage display library.	The isolated antibody clones were characterised based on their gene sequences and binding characteristics. Three clones representing unique gene families (clone 48: IgHV3-LV1; clone 49: IgHV3-LV3; clone 50: IgHV6-LV3) were isolated, but only clones 48 and 49 showed successful insertion of the full-length scFv antibody sequence after sub-cloning. The antibody clones that were isolated were analysed and described using their genetic sequences and their ability to bind to the target. Three clones, each representing distinct gene families (clone 48: IgHV3-LV1; clone 49: IgHV3-LV3; clone 50: IgHV6-LV3), but only clones 48 and 49 exhibited successful insertion of the complete scFv antibody sequence following sub-cloning.	Both monoclonal antibodies exhibited a high level of specificity and sensitivity towards the target antigen. The ELISA assay demonstrated the diagnostic capability of the monoclonal antibodies. These proteins can also be valuable for investigating interactions between hosts and parasites as well as for therapeutic purposes.
Trashin S et al., 2021, Belgium [64]	A time-efficient sandwich immunosensor using nanobodies (small recombinant single-domain antibodies) originating form camelid heavy chain-only antibodies to detect *T. canis* antigens.	This test had a high sensitivity, detecting levels as low as pg/mL by using a redox cycle consisting of a photocatalytic oxidation and electrochemical reduction steps.	The authors report that this approach surpasses assays using ordinary antibodies by a factor of at least two orders of magnitude, indicating potential for electrochemical immunoassays targeting difficult-to-detect low quantities of antigens.
Morales-Yánez F et al., 2020, Belgium [65]	Nanobody-based electrochemical magnetosensor to be used for superior detection of *Toxocara* excretory/secretory (TES) antigens. A bivalent biotinylated nanobody was used as a capturing agent on the surface of paramagnetic beads, precoated with streptavidin. A horseradish peroxidase marked antibody was employed for detection.	A total of 87 samples were tested, with 33 being detected positive for the TES antigen (38%), using the electrochemical magnetosensor assay.	This nanobody-based electrochemical allows for highly sensitive quantification of TES antigens in serum and could be used for the diagnosis of active human toxocarosis.
Morales-Yánez F et al., 2019, Belgium [66]	Quantitative *Toxocara* excretory/secretory (TES) antigen assay, to be used for diagnosing active cases of human toxocarosis. Nanobodies, single domain antigen binding fragments originating from camelid heavy chain-only antibodies, are employed for high specificity. An electrochemical magnetosensor with an amperometric read-out is used for high sensitivity results.	TES antigen could reliably be detected at concentrations as low as 10 pg/mL in phosphate-buffered saline and 30 pg/mL in serum.	The authors consider this method to be the most sensitive TES quantitative assay tested up to this point. It could be employed to develop point of care diagnostic systems and expanded to other conditions, where high sensitivity and specificity are required. This nanobody-based assay is especially important as it detects active infection.
Molecular methods			
Zibaei M et al., 2022, Iran [67]	This study determined the expression levels of circulating miRNA 21 and miRNA 103a as possible biomarkers for predicting and diagnosing toxocarosis in Wistar rats infected with *T. canis.* Serum samples were collected from the 30 Wistar rats for 60 days after infection with 2500 *T. canis* eggs. Plasma samples were obtained and used for quantitative real-time PCR (qPCR) tests to measure the transcription levels of miRNA 21 and miRNA 103a.	Anti-*Toxocara* IgG was found in 23.3% of the infected rats, specifically in 7 out of 30. Analysis of miRNAs 21 and 103a at the molecular level revealed that the expression levels of these miRNAs were equivalent in both the Toxocara-positive and negative sample groups, with no significant correlation.	The results of this study indicate that miRNAs 21 and 103a have significant potential as biomarkers and diagnostic tools for toxocarosis. Nevertheless, the alterations in the expression of these miRNAs were insufficient to serve as diagnostic biomarkers.
Avila HG et al., 2021, Argentina [68]	The authors conceived a new coprological loop-mediated isothermal amplification (LAMP) assay for the simultaneous detection of *T. canis* and *T.* cati. The primer targeted a specific area of the mitochondrial cox-1 gene. The amplification conditions were assessed at different temperatures and time lengths using varied concentrations of malachite green dye. The analytical sensitivity was assessed by doing serial dilutions of genomic DNA obtained from adult worms of *T. canis* and *T. cati* as well as serial dilutions of DNA isolated from faeces using an inexpensive in-house approach.	The LAMP assay was used on faecal samples from an area where the disease is common, being able to detect very small amounts of DNA (10–100 femtograms) and diluted DNA (10^−5^) retrieved from the faeces. The assay had a specificity of 100%, accurately identifying the target DNA without any false positives.	This affordable and innovative technique can be used to identify the predominant causative agents of toxocarosis in regions where the disease is prevalent. It allows for the implementation of prevention methods in low-resource settings.
Azimian H et al., 2021, Iran [69]	By using the loop-mediated isothermal amplification (LAMP) technique, the authors aimed to estimate the molecular prevalence of *Toxocara* species in stray cats. Toxocara eggs were separated from 95 stool samples by a flotation method. Microscopic analysis was conducted following the separation and extraction of the supernatants. The LAMP reaction was performed utilising the internal transcribed spacer 2 (ITS2) gene primers specific to *Toxocara* species, along with a suitable master mix.	Upon microscopic analysis, it was determined that 19 stool samples tested positive for *Toxocara*. The same 19 positive samples were likewise confirmed as positive using the LAMP technique.	Preventive measures, such as sterilising stray cats, should be implemented to control their proliferation and protect public spaces from contamination. The LAMP approach, which is both simple and extremely accurate, could distinguish between distinct *Toxocara* species in animals.
Moura MQ et al., 2020, Brazil [70]	In this study, a quantitative real-time polymerase chain reaction (qPCR) method was employed to detect and measure the amount of *T. canis* parasites in the mouse brain. A total of 24 mice were allocated into six groups. Among these groups, five were exposed to varying doses of 1000, 500, 250, 100 and 50 *T. canis* larvae, while the sixth group served as an uninfected control.	Autopsies were conducted 45 days after infection to retrieve the brain, from which two 20 mg tissue samples were used for DNA extraction and analysis. The remaining brain tissue was digested to determine the larval count using microscopy. The quantity of DNA copies was determined using the standard DNA quantification curve (E = 93.4%, R2 = 0.9655 and Y = −3.415).	The findings of this paper validate the utility of the qPCR method as a valuable means of identifying and measuring *T. canis* DNA in murine hosts, even in cases where the tissues of the animals harbour only a small number of parasites. This study can be helpful in developing a sensitive qPCR test for human biopsy sample analysis.
Özbakış G et al., 2019, Turkey [71]	Molecular detection of *Toxocara* larvae in liver, muscle, lung and brain tissue. A group of 24 BALB/c mice were exposed to 1000 embryonated *T. canis* eggs. Autopsies were conducted on days 2, 4, 7 and 14 after infection. Some of the samples were treated with pepsin–HCl, while the molecular technique was employed for the remaining samples to duplicate the mitochondrial DNA adenosine triphosphate (ATP) synthase subunit-6 gene area of *T. canis*.	PCR analysis had a sensitivity/accuracy of 83.3%/88.8% for liver, 87.5%/91.6% for lung and 75.0%/83.3% for the brain, forelimb and hindlimb muscle samples.	While the technique was conceived and applied for BALB/c mice tissues, it is possible that it can also be employed in other non-permissive infected hosts and materials contaminated with *T. canis*.

## Data Availability

No new data were created or analyzed in this study. Data sharing is not applicable to this article.
